# Veterinary intelligence: integrating zoonotic threats into global health security

**DOI:** 10.1177/01410768211035355

**Published:** 2021-08-05

**Authors:** Gemma Bowsher, Tracey McNamara, Rose Bernard, Richard Sullivan

**Affiliations:** 1 Conflict and Health Research Group, King’s College London, London WC2R 2LS, UK; 2 Western University of Health Sciences College of Veterinary Medicine, Pomona, CA 91766, USA

## Animal diseases are human diseases

Zoonotic diseases are leading threats to public health globally. The recent G7 meeting of world leaders made strong political commitments to strengthening One Health approaches at the human–animal interface as an integral element of the global health security architecture. Repeated epidemics and pandemics from Ebola to COVID-19 have demonstrated the systematic disregard of zoonotic disease within what still remains a predominantly human-centric public health approach. In particular, commitments to the expansion of pathogen surveillance and health intelligence require the development of novel approaches to improve and strengthen our domestic capabilities for species neutral monitoring, which requires the sustained involvement of veterinary colleagues.

The belief that medical and veterinarian communities should be synergistic collaborators in outbreak response has been largely neglected.^1^ At present, there is a dearth of systems capable of providing indicators and warnings for zoonotic diseases beyond livestock management (e.g. bovine TB, foot and mouth, etc.). Companion (e.g. domesticated dogs and cats), zoo and shelter animals exist in close proximity to human populations and with limited syndromic monitoring in place, remain an under-evaluated, but potentially high-risk disease reservoir for potential zoonoses. SARS-CoV-2 virus with its ability to move in and out of multiple mammalian species including humans has shown us that new approaches are necessary to detect high consequence pathogens in the zoonotic space. Health security intelligence requires the integration of veterinary analysts within a multi-source threat intelligence framework that incorporates an ‘all-species’ approach. Furthermore, domesticated animals in high-income countries are as much a potential reservoir of high-threat zoonoses as the oft-cited wildlife in wet markets or equatorial rainforests. Veterinary intelligence thus needs to cover a complex global ecosystem from the very remote to the local high street and zoo.

## The impact of animal disease on humans

Human–animal interactions are a regular source of disease outbreaks, particularly in mammalian species.^2^ A multitude of factors from climate change, population displacement into novel ecosystems, social norms such as possession of companion animals and husbandry practices are radically expanding the contact surface and evolutionary pressures for zoonotic pathogens. From Ebola, to MERS, to SARS-CoV-2, animal reservoirs abiding in close proximity to human populations generate the majority of our high consequence infectious diseases that have led to nearly 1500 epidemics and pandemics across all countries in the last decade.^3^ A false distinction has been made in traditional public health approaches between human and animal disease threats. Nations such as the UK, USA and Australia maintain strict livestock public health systems; however, the broader landscape of relapsing-remitting infectious disease interactions between animals and humans at all scales of analysis has not been integrated effectively into domestic pandemic threat preparedness.

It is well-documented that shelter animals in particular are high-risk populations given their high levels of stress and susceptibility to infectious pathogens. A 2017 outbreak of H7N2 bird flu in the New York’s feline shelter population demonstrated novel transmission pathways across a large population of over 300 animals and into people. Previously identified in the city’s poultry markets, the H7N2 virus had not been known to cross over into feline species prior to this event.^4^ Given absent epidemiological surveillance in non-shelter companion animals, the pathway of transmission into domesticated felines during this episode remains an open question. SARS-CoV-2 too has illustrated an array of unknowns regarding the inter-species transfer of pandemic-potential pathogens. The News of SARS-CoV-2 infection in the Danish mink population has resulted in the culling of captive mink given evidence of intra-population transfer, and potential mink-to-human transmission.^5^ The virus has also been identified occurring naturally in other species including dogs, cats, tigers and lions, while other species proven to be susceptible to SARS-CoV-2 in experimental conditions include Syrian hamsters, macaques, fruit bats and ferrets.^6^ Hong Kong instituted a mandatory quarantine for all dogs of people who tested positive for the virus following a positive test and subsequent death of a 17-year-old Pomeranian.^7^ The precautionary approach employed by the Hong Kong authorities highlights a number of knowledge gaps in our present systems for detecting early warning signals in companion animals.

## Animal surveillance: indicators and warnings

In the UK and USA there are no compulsory systems for real-time situational awareness of emerging pathogens in veterinary populations. In an urban setting, for example, veterinary practice networks have limited analytical and visualisation platforms with real-time systems for detection of infectious disease signal above baseline and there is no population health surveillance for companion animals. Lack of funding and governance frameworks contribute in equal measure. Testing is voluntary in companion animals, and consequently requires funding from either individuals or insurance providers. The UK-based SAVSNET, a voluntary syndromic surveillance platform operating across 301 veterinary practices was recently used to detect and evaluate an outbreak of canine gastroenteritis following an initial request for information spotted by a veterinarian on Facebook.^8^ This intervention, which coupled syndromic data from SAVSNET with the rapid deployment of field epidemiology and genomic testing, shows the potential for early detection of emerging pathogens in susceptible veterinary populations. But this also reflects the *ad hoc* nature of warnings which have manifested during the ongoing COVID-19 pandemic.

SARS-CoV-2 has been identified in tigers and lions, with the index case in New York thought to be as a result of cross-infection from an asymptomatic zoo-keeper.^9^ Unlike the USA, animals who die in zoos in the UK do not undergo mandatory necropsy, missing a crucial opportunity to detect potential and confirmed disease present in animal populations. Zoos use sentinel flocks to produce signal detection for emerging pathogens; however, the unexpected transmission pathways observed during the COVID-19 pandemic and previous outbreaks raises the question of whether regular testing of captive animals in zoos, shelters and household settings would offer an effective signal detection mechanism to produce more effective intelligence regarding viral dynamics across all populations. Genotyping for variants of concern in human populations during the COVID-19 pandemic has shown the application of these approaches in public health measures; however, a limited focus has been directed towards genomic surveillance at the animal–human interface. Reports of SARS-CoV-2 variants of concern implicated in animal disease are emerging more frequently in the literature,^10,11^ and the range of unknowns regarding variants of concern and their role in cross-species transmission merits further study.

Indicators and warnings for zoonotic surveillance can also utilise the growing repertoire of open source and signals intelligence approaches. For example, in 2016 National Aeronautics and Space Administration employed the Goddard Earth Sciences and Technology Center to evaluate climate disturbances using meteorological satellites to predict flooding patterns, vegetation cover and sea surface temperatures.^12^ This method was used to generate a surveillance system integrating diverse disease-favouring conditions which successfully alerted researchers to the imminent emergence of Rift Valley Fever in Kenyan livestock populations six weeks in advance. Rift Valley Fever possesses significant animal-human transmissibility and in 2006–2007 infected over 200,000 people across Eastern Africa, killing approximately 500. Swedish authorities similarly used real time satellite-derived data and oceanographic records to identify an emerging *Vibrio* outbreak.^13^ The variety of multi-open-source systems, combined with well-trained analysts has the potential to produce high-quality, rapidly actionable zoonotic outbreak intelligence; however, few if any countries have this capability integrated into pandemic operations.^14^ The generation of high-quality zoonotic situational awareness at the earliest possible stages of an outbreak must be a priority for future health security systems, in particular those aligned with the G7’s One Health imperatives that integrate environment–animal–human domains.

## Veterinary intelligence

We have previously described the need to transition from traditional public health/biosurveillance to a health security intelligence approach to epi-pandemics.^15–17^ This also needs to include zoonotic threats through a veterinary intelligence approach. The repeated interface of humans and animals necessitates a focus on generating data and producing actionable intelligence to aid the prevention and early detection of zoonotic spillover events. Priority intelligence requirements for the veterinary sciences are the delineation of emerging threats from companion, livestock and wildlife communities, which require the integration of early warning tools, open-source platforms, multi-source and multi-species surveillance, proactive diagnostics, field testing technologies and increased attention to necropsy in captive and wild animals. The anticipation and early detection of potential zoonotic events should be a first-order objective for any developing health security agenda in both global and domestic settings.

Funding in this area is a challenge, with extant surveillance systems in livestock or equine populations existing primarily for reasons of commercial significance or due to international trade and travel regulations. Governments and public health systems have repeatedly declined to fund broader veterinary populations including companion animals, resulting in our current limited capabilities for rapid situational awareness. Investments from the private and/or governmental sectors to develop the required multi-source all-species platforms are vital to building a robust health security intelligence architecture that integrates veterinary intelligence. A further dimension relevant to the monitoring of the human–animal interface is the role of veterinarians as a high-risk population most likely to encounter novel and emerging zoonoses. Implementing surveillance measures across at risk veterinary groups utilises both their value for early warning generation, and introduces improved opportunities for integrated occupational and public health interventions.

## Conclusion

The siloing of veterinary and medical communities obstructs the development of an effective health security research agenda and training pathways that promote collaboration and synergistic working in these domains. Integration, not only within a One Health agenda, but in a systematised health security intelligence framework opens up horizons for a more holistic disease preparedness system, able to detect and respond to an array of infectious disease threats from novel viruses to AMR whether they emerge in animals or humans. Ignoring the potential for animal infections to produce and propagate human disease is a failure of health security. Zoonoses continue to pose the greatest health security threat to human and animal populations alike; effective future epi-pandemic preparedness demands improved systems for ‘species neutral’ health security intelligence.

## Declarations

**Ethics approval:** Not required.

**Guarantor:** GB.

**Contributorship:** GB wrote the first draft and undertook multiple rounds of editing. TM, RB and RS contributed significant conceptual development and analytic insights, as well as collaborating on revising and preparing the manuscript for submission.
